# Van Wyk–Grumbach Syndrome With Insulin Resistance in Children: A Case Report

**DOI:** 10.1002/ccr3.71527

**Published:** 2025-11-28

**Authors:** Hui Liu, Gaojie Liu, Hui Wang, Weicai Suo, Yizhong Wang, Hongfang Ding

**Affiliations:** ^1^ Department of Pediatrics Shengli Oil Field Central Hospital Dongying Shandong China; ^2^ Department of Gastroenterology, Hepatology, and Nutrition, Shanghai Children's Hospital, School of Medicine Shanghai Jiao Tong University Shanghai China; ^3^ Gut Microbiota and Metabolic Research Center, Institute of Pediatric Infection, Immunity and Critical Care Medicine, School of Medicine, Shanghai Jiao Tong University Shanghai China

**Keywords:** hypothyroidism, insulin resistance, precocious puberty, TSH, VWGS

## Abstract

This case reports an 8 year‐5 month‐old girl diagnosed with Van Wyk–Grumbach Syndrome (VWGS) and co‐existing insulin resistance from China. The patient achieved complete resolution of clinical manifestations with thyroid hormone replacement therapy. Pediatricians should raise awareness of this rare and underdiagnosed disorder to avoid unnecessary tumor investigations or surgical intervention.

## Graphical abstract

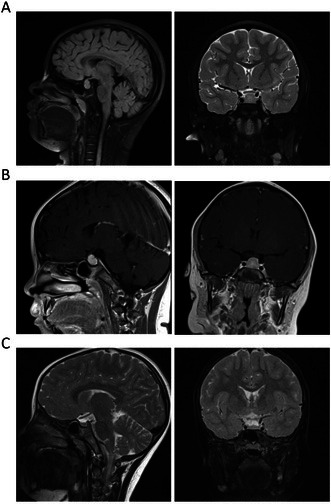

**Abbreviations**
BMI: body mass index
FINS: fasting insulin
FPG: fasting blood glucose
FSH: follicle‐stimulating hormone
GLUT4: glucose transporter type‐4
HOMA‐IR: homeostasis model assessment of insulin resistance
IRE1α: inositol‐requiring enzyme 1α
MRI: magnetic resonance imaging
PCOS: polycystic ovary syndrome
PRL: prolactin
Tg: thyroglobulin
TLR4: toll‐like receptor 4
TPO: thyroid peroxidase
TSH: thyroid‐stimulating hormone
VWGS: Van Wyk–Grumbach Syndrome
XBP‐1: spliced X‐box binding protein 1

## 1 Introduction

Van Wyk–Grumbach Syndrome (VWGS) in children is a rare presentation of untreated hypothyroidism characterized by clinical features of peripheral precocious puberty, enlarged multicystic ovaries, and delayed bone age [1]. The precocious puberty in VWGS is isosexual and mediated by a high level of thyroid‐stimulating hormone (TSH)‐induced follicle‐stimulating hormonal response by activating follicle‐stimulating hormone (FSH) receptor [2]. The affected girls commonly present with short stature with delayed bone age, vaginal bleeding, galactorrhoea, breast development, and multicystic ovaries, whereas affected boys typically have macroorchidism and penile enlargement without other signs of virilization, and axillary and pubic hair are absent in both girls and boys with VWGS [3]. Other manifestations are also reported in the literature, including pituitary enlargement, severe anemia, ascites, ovarian torsion, intracranial mass effect, acute surgical abdomen, Cullen's sign, and positivity of tumor markers [4]. Given the high heterogeneity in its presentation, early and accurate diagnosis is critical for the management of children with VWGS to avoid unnecessary investigations for tumors or surgical intervention. The diagnosis of VWGS is based on long‐standing hypothyroidism with significantly elevated levels of TSH, as well as the imaging techniques, such as magnetic resonance imaging (MRI). Currently, thyroid hormone replacement is the optimal therapy for VWGS, which can resolve symptoms rapidly and reverse to the prepubertal state [4].

Here, we report a case of VWGS presented with typical features of severe hypothyroidism, breast development, delayed bone age, short stature, overweight, pituitary enlargement, elevated serum prolactin level, and co‐existing insulin resistance in an 8 year‐5 month‐old girl from China. To the best of our knowledge, this study is the first to report a VWGS case accompanied by insulin resistance, which further expands its clinical spectrum.

## 2 Case Presentation

An 8 year‐5 month‐old girl of Han ethnicity was admitted because of quick weight gain, breast development, and short stature. She was born normally, intellectually normal, and previously healthy. She is the second child of a healthy nonconsanguineous couple, and the family history was unremarkable. The mother's height is 168 cm, the father's height is 178 cm, and her 22 year‐old brother is 180 cm.

On admission, physical examination showed that she was 123 cm tall (3rd–10th percentile), weighed 40 kg (> 97th percentile), and had a body mass index (BMI) of 26.4 kg/m^2^ (> 97th percentile). Her body temperature was 36.7°C, heart rate was 82 beats per minute, respiratory rate was 26 breaths per minute, and blood pressure was 109/79 mmHg. The cardiopulmonary and abdominal examinations were normal, and the muscle strength and tension of the limbs and the neurological examinations were unremarkable. She had a significant increase in weight (approximately 10 kg) in the past year. Breast development was at Tanner's stage 3, without growth of pubic and axillary hair. She had dry skin with no rash or hemorrhagic spots, and pigmented skin was observed in the cervical region and armpit.

**FIGURE 1:**
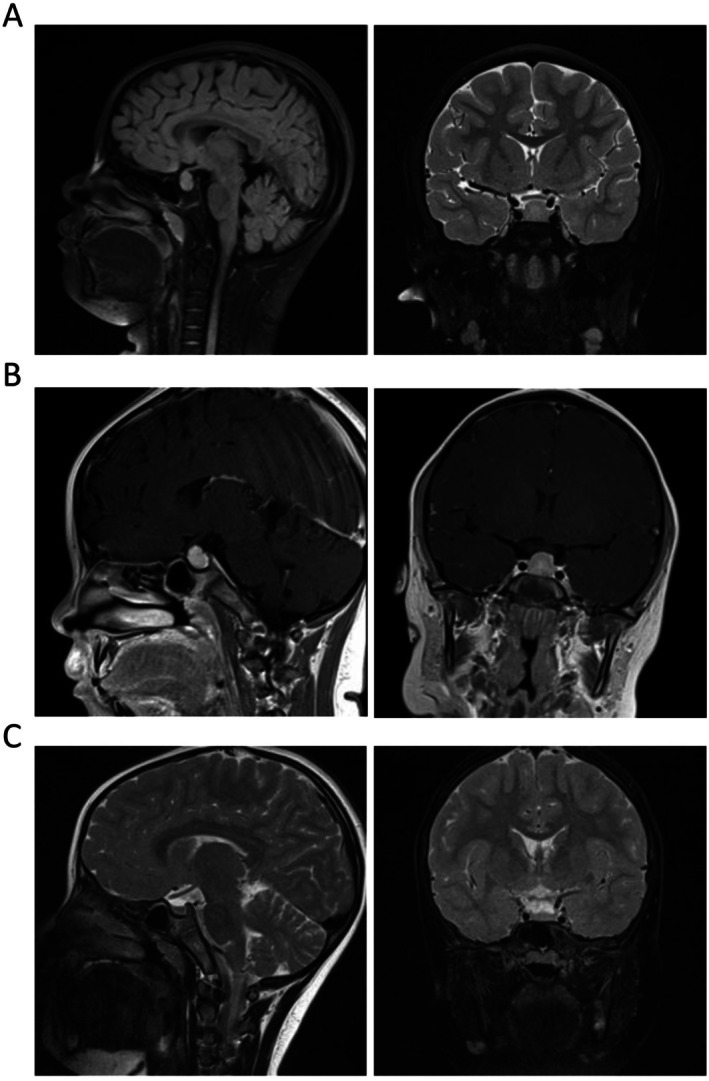
Brain magnetic resonance imaging (MRI) at diagnosis and after 3 months of levothyroxine replacement. (A) Pituitary hyperplasia at diagnosis (craniocaudal diameter: 13.5 mm, slightly compressing the optic chiasm). (B) Enhanced MRI with pituitary slices excluded gigantism and pituitary adenoma as a cause for early puberty. (C) Normal pituitary size after 3 months of levothyroxine replacement (craniocaudal diameter: 5.2 mm).

As shown in Table 1, laboratory tests revealed highly elevated serum levels of TSH (> 100 μIU/mL, normal range: 0.70–4.77 μIU/mL), prolactin (PRL, 60.85 ng/mL, normal range: 3.77–19.78 ng/mL), and fasting insulin (FINS, 302.70 pmol/L, normal range: 13–161 pmol/L). The serum fasting blood glucose (FPG, 5.24 mmol/L, normal range: 3.89–6.11 mmol/L) was normal. The homeostasis model assessment of insulin resistance (HOMA‐IR) value was 10.15. The levels of thyroid peroxidase (TPO) antibodies (23.20 IU/mL, normal range: < 9 IU/mL) and thyroglobulin (Tg) antibodies (11.70 IU/mL, normal range: < 4 IU/mL) were increased. Cortisol and low‐density lipoprotein cholesterol were slightly elevated, while low serum levels of free thyroxine (T4, 0.46 ng/mL, normal range: 0.89–1.37 ng/mL) and thyroglobulin (Tg, < 0.10 ng/mL, normal range: 1.15–130.77 ng/mL) were observed. FSH, estradiol, and luteinizing hormone levels were normal, and the GnRH stimulation test was negative. Bone age revealed from a left‐hand X‐ray was delayed 2.5 years with respect to chronological age. An abdominal ultrasound showed a normal uterus and both ovaries. Ultrasonography revealed a mildly reduced volume and an inhomogeneous echo pattern of the thyroid gland. An MRI of the brain indicated pituitary enlargement (Figure 1A), and pituitary adenoma was excluded by an enhanced MRI (Figure 1B). Thus, the patient was diagnosed with VWGS with insulin resistance.

**TABLE 1:** Laboratory findings at diagnosis and at 3 month follow‐up.

Laboratory parameter	Normal range	At diagnosis	After 1 month	After 3 months
TSH (μIU/mL)	0.70–4.77	> 100	0.980	6.168
Free T4 (ng/dL)	0.89–1.37	0.46	1.62	1.05
Free T3 (pg/mL)	2.79–4.42	2.80	7.55	3.86
Total T4 (μg/dL)	6.16–10.32	2.46	15.87	10.08
Total T3 (ng/mL)	1.13–1.89	1.16	2.45	1.36
TPO antibodies (IU/mL)	< 9	23.20	15.80	13
Tg antibodies (IU/mL)	< 4	11.70	6.60	3.4
Tg (ng/mL)	1.15–130.77	< 0.10	0.10	/
TR antibodies (IU/L)	0–1.75	0.59	0.51	0.61
LH (mIU/mL)	0.04–2.18	< 0.20	< 0.2	/
FSH (mIU/mL)	0.80–8.41	2.41	1.46	/
E2 (pg/mL)	0–74.5	< 20	/	/
PRL (ng/mL)	3.77–19.78	60.85	18.20	/
FINS (pmol/L)	13–161	302.70	39.89	38.52
FPG (mmol/L)	3.89–6.11	5.24	4.12	4.58
HOMA‐IR	< 2.5	10.15	1.05	1.13
HbA1c (%)	< 6%	5.8%	/	5.4%
Cortisol (μg/dL)	4.26–24.85	25.182	8.956	/
ACTH (pg/mL)	7.2–63.4	51.10	/	/
TC (mmol/L)	3.10–5.69	6.54	5.62	/
TG (mmol/L)	0.56–1.7	0.9	0.8	/
HDL‐C (mmol/L)	0.77–2.25	1.35	1.40	/
LDL‐C (mmol/L)	1.27–4.13	4.65	4.02	/

Abbreviations: ACTH, adrenocorticotropic hormone; E2, estradiol; FINS, fasting insulin; FPG, fasting blood glucose; HbA1c, glycated hemoglobin; HDL‐C, TPO, thyroid Peroxidase; high‐density lipoprotein cholesterol; HOMA‐IR, FSH, follicle‐stimulating hormone; homeostasis model assessment of insulin resistance; LDL‐C, low‐density lipoprotein cholesterol; LH, luteinizing hormone; PRL, prolactin; T3, triiodothyroxine; T4, thyroxine; TC, total cholesterol; Tg, thyroglobulin; TG, triglyceride; TR, thyroxine receptor; TSH, thyroid‐stimulating hormone.

## 3 Conclusions and Results (Outcome and Follow‐Up)

After the diagnosis, the patient was initially treated with levothyroxine at 50 μg once daily. After 1 month of treatment, all laboratory parameters were back to the normal range (Table 1). A brain MRI showed a normal pituitary size at the 3 month follow‐up (Figure 1C). The patient was managed with continuous levothyroxine treatments and monitored closely in the following 5 years, and all clinical manifestations were resolved with normal growth and development. At this writing, she is 14 years old with a height of 165 cm (> 90th percentile).

## 4 Discussion

This study reported an 8 year‐5 month‐old girl diagnosed with VWGS accompanied by insulin resistance. The patient presented with short stature, overweight, dry skin, and Tanner's stage 3 breast development, without growth of pubic and axillary hair. A hormonal assay revealed high serum levels of TSH and PRL. In consistency with previous reports [5, 6, 7], the serum T4 level was low. Increased serum levels of TPO antibodies and Tg antibodies were suggestive of autoimmune hypothyroidism [8]. Leonardi et al. reported a young girl with VWGS due to severe Hashimoto thyroiditis who presented with rhabdomyolysis [9]. Similarly, X‐ray indicated delayed bone age of 2.5 years, and pituitary enlargement was observed by a brain MRI, but no abnormalities of the uterus and both ovaries were observed in the patient. Unexpectedly, FINS was highly elevated in our patient, which made a diagnosis of insulin resistance according to the HOMA‐IR value. Such a case of VWGS, along with insulin resistance, to the best of our search in the literature, has never been reported previously.

The pathophysiology of VWGS is speculated to be associated with the activation of ovarian FSH receptor (FSH‐R) by high concentrations of TSH due to the molecular similarities between FSH and TSH (sharing a common alpha subunit) [10]. FSH‐R activation results in ovarian hyperstimulation and oversecretion of gonadotropins, leading to precocious puberty [10]. Almost all the reported VWGS cases presented with multicystic ovaries, attributed to the markedly increased TSH levels [11]. Unexpectedly, our patient did not have abnormal ovaries, unlike the other cases in the literature [11]. In 2014, Christens et al. reported that another 8 year‐old girl with VWGS did not have breast development or multicystic ovaries, which may be due to early recognition and relatively low TSH levels [2]. Interestingly, the serum levels of FSH, estradiol, and luteinizing hormone were not elevated in our patient, which may be related to the clinical features of isolated breast development without enlarged ovaries and uterus. Therefore, VWGS should be kept in mind even in individuals without clear ovarian involvement. Although the patient had a high serum level of PRL, galactorrhea did not occur. It has been reported that hyperprolactinemia is associated with pituitary stalk compression due to pituitary enlargement‐induced disruption of hypothalamic inhibition, or thyrotropin‐releasing hormone (TRH)‐related hyperprolactinemia [12]. PRL makes the ovary sensitive to circulating gonadotropin and accelerates follicular maturation [13]. In addition, skin pigmentation is not common in hypothyroidism, which may be due to the homology or cross‐reactivity between TSH and melanocyte‐stimulating hormone (MSH) [14]. Inconsistent with previously reported VWGS cases, complete resolution of clinical manifestations was achieved in our patient after 3 months of thyroid hormone replacement therapy.

The association of thyroid disorders and insulin resistance has been reported in the literature, including both hyperthyroidism and hypothyroidism [15]. Thyroid hormones are involved in glucose metabolism and insulin resistance [16]. Kar et al. showed that the incidence rate of insulin resistance was significantly higher in patients with hypothyroidism than in control subjects [17]. Amrousy et al. reported that the levels of FINS, HOMA‐IR value, and leptin were significantly elevated in hypothyroid children, particularly in hypothyroid children with obesity [18]. Furthermore, other conditions associated with hypothyroidism or subclinical hypothyroidism are related to insulin resistance. For example, type 2 diabetes mellitus patients with hypothyroidism showed increased metabolic disorder, poor glycemic control, and insulin resistance [19]. Polycystic ovary syndrome (PCOS) patients with subclinical hypothyroidism had significantly higher HOMA‐IR and FINS levels than those without subclinical hypothyroidism [20]. Although insulin resistance was not described in children with VWGS previously, it is likely caused by hypothyroidism in our patients. On the contrary, a recent study reported that subclinical hypothyroidism did not lead to insulin resistance in children [21]. Nevertheless, further studies are needed to investigate the relationship between VWGS and insulin resistance.

Although the molecular mechanisms of insulin resistance in patients with the hypothyroid state were not fully understood, several studies have shown that impaired insulin sensitivity in subclinical hypothyroidism is primarily attributed to decreased glycogen synthesis, inhibition of glucose transporter type‐4 (GLUT4) translocation, and reduced intracellular glucose utilization [20, 22]. The inositol‐requiring enzyme 1α (IRE1α) and spliced X‐box binding protein 1 (XBP‐1) pathway activation induced hepatic endoplasmic reticulum stress has been implicated in leading to insulin resistance in hypothyroid patients [23]. Hypothyroid‐associated chronic low‐grade inflammation can impair insulin signaling pathways, such as TSH activating toll‐like receptor 4 (TLR4)‐mediated inflammatory pathways [24]. Furthermore, Zhang et al. recently reported that thyrotropin exacerbated insulin resistance by triggering macrophage inflammation in subclinical hypothyroidism [25].

In summary, the current study described an 8‐year‐5‐month‐old girl with typical clinical features of VWGS and co‐existing insulin resistance from China. Given the syndrome's heterogeneity and diverse clinical presentations, pediatricians should raise awareness of this rare and underdiagnosed disorder to avoid unnecessary tumor investigations or surgical interventions.

## Author Contributions

**Hui Liu:** data curation, investigation, writing – original draft, writing – review and editing. **Gaojie Liu:** data curation, investigation, writing – original draft, writing – review and editing. **Hui Wang:** formal analysis, methodology. **Weicai Suo:** formal analysis, methodology. **Yizhong Wang:** conceptualization, funding acquisition, supervision, validation, writing – review and editing. **Hongfang Ding:** conceptualization, data curation, formal analysis, writing – review and editing.

## Funding

This work was supported by the National Natural Science Foundation of China, 32370951.

## Consent

Written informed consent was obtained from the patient for the publication of this case report.

## Conflicts of Interest

The authors declare no conflicts of interest.

## Data Availability

The data that support the findings of this study are available from the corresponding author upon reasonable request.

## References

[1] J. J. Van Wyk and M. M. Grumbach , “Syndrome of Precocious Menstruation and Galactorrhea in Juvenile Hypothyroidism: An Example of Hormonal Overlap in Pituitary Feedback,” Journal of Pediatrics 57, no. 3 (1960): 416–435.

[2] A. Christens , L. Sevenants , J. Toelen , D. Bullens , and K. Casteels , “Van Wyk and Grumbach Syndrome: An Unusual Form of Precocious Puberty,” Gynecological Endocrinology 30, no. 4 (2014): 272–276. 24568556 10.3109/09513590.2013.871523

[3] S. Zhang , J. Yang , R. Zheng , L. Jiang , Y. Wei , and G. Liu , “VanWyk‐Grumbach Syndrome in a Male Pediatric Patient: A Rare Case Report and Literature Review,” Experimental and Therapeutic Medicine 13, no. 3 (2017): 1151–1154. 28450956 10.3892/etm.2017.4086 PMC5403655

[4] A. A. Arellano‐Llamas , A. Hernandez‐Caballero , E. Delgado‐Mendoza , and M. A. Catalan‐Ruiz , “Van Wyk‐Grumbach Syndrome and Gonadectomy,” Children (Basel) 11, no. 7 (2024): 831. 39062280 10.3390/children11070831 PMC11274782

[5] M. Biswas , M. K. Sinha , M. K. Das , and S. Sarkar , “Van Wyk‐Grumbach Syndrome With Hemangioma in an Infant,” Journal of Pediatric Endocrinology and Metabolism 31, no. 9 (2018): 1057–1060. 30028725 10.1515/jpem-2018-0049

[6] D. Sivasubramanian , V. Senthilkumar , S. Aravind , A. R. Rajasekar , S. Prasaanth , and S. Sanil , “Van Wyk‐Grumbach Syndrome With Bilateral Inguinal Hernia: A Case Report,” International Journal of Surgery Case Reports 127 (2025): 110975. 39884170 10.1016/j.ijscr.2025.110975 PMC11815951

[7] S. Kusuma Boddu , A. Ayyavoo , V. Hebbal Nagarajappa , K. V. Kalenahalli , S. Muruda , and R. Palany , “Van Wyk Grumbach Syndrome and Ovarian Hyperstimulation in Juvenile Primary Hypothyroidism: Lessons From a 30‐Case Cohort,” Journal of the Endocrine Society 7, no. 6 (2023): bvad042. 37197410 10.1210/jendso/bvad042 PMC10184442

[8] A. Magrini , A. Pietroiusti , L. Coppeta , et al., “Shift Work and Autoimmune Thyroid Disorders,” International Journal of Immunopathology and Pharmacology 19, no. 4 (2006): 31–36. 17291404

[9] A. Leonardi , L. Penta , M. Cofini , L. Lanciotti , N. Principi , and S. Esposito , “Rhabdomyolysis in a Young Girl With Van Wyk‐Grumbach Syndrome due to Severe Hashimoto Thyroiditis,” International Journal of Environmental Research and Public Health 15, no. 4 (2018): 704. 29642533 10.3390/ijerph15040704 PMC5923746

[10] J. N. Anasti , M. R. Flack , J. Froehlich , L. M. Nelson , and B. C. Nisula , “A Potential Novel Mechanism for Precocious Puberty in Juvenile Hypothyroidism,” Journal of Clinical Endocrinology and Metabolism 80, no. 1 (1995): 276–279. 7829625 10.1210/jcem.80.1.7829625

[11] Y. Komatsu and N. Kikuchi , “Van Wyk‐Grumbach Syndrome: A Case Report and Review of the Literature,” Clinical Pediatric Endocrinology 34, no. 4 (2025): 268–274. 41049522 10.1297/cpe.2025-0032 PMC12494393

[12] C. R. Nicolescu , L. Bazus , and J. L. Stephan , “Severe Acquired Hypothyroidism and Van Wyk‐Grumbach Syndrome in Two Children,” Case Reports in Pediatrics 2024 (2024): 8919177. 39015673 10.1155/2024/8919177 PMC11251785

[13] T. L. Copmann and W. C. Adams , “Relationship of Polycystic Ovary Induction to Prolactin Secretion: Prevention of Cyst Formation by Bromocriptine in the Rat,” Endocrinology 108, no. 3 (1981): 1095–1097. 7460836 10.1210/endo-108-3-1095

[14] M. Kruszynski , G. Kupryszewski , U. Ragnarsson , et al., “TRH Analogue With C‐Terminal Thioamide Group. Synthesis, Receptor Binding, TSH‐Releasing Activity and Alpha‐MSH‐Releasing Activity,” Experientia 41, no. 12 (1985): 1576–1577. 3000815 10.1007/BF01964815

[15] M. Gierach , J. Gierach , and R. Junik , “Insulin Resistance and Thyroid Disorders,” Endokrynologia Polska 65, no. 1 (2014): 70–76. 24549605 10.5603/EP.2014.0010

[16] D. A. Mendez and R. M. Ortiz , “Thyroid Hormones and the Potential for Regulating Glucose Metabolism in Cardiomyocytes During Insulin Resistance and T2DM,” Physiological Reports 9, no. 16 (2021): e14858. 34405550 10.14814/phy2.14858 PMC8371345

[17] K. Kar and S. Sinha , “Variations of Adipokines and Insulin Resistance in Primary Hypothyroidism,” Journal of Clinical and Diagnostic Research 11, no. 8 (2017): BC07–BC09. 28969110 10.7860/JCDR/2017/26666.10345 PMC5620750

[18] D. El Amrousy , D. El‐Afify , and S. Salah , “Insulin Resistance, Leptin and Adiponectin in Lean and Hypothyroid Children and Adolescents With Obesity,” BMC Pediatrics 22, no. 1 (2022): 245. 35501770 10.1186/s12887-022-03318-x PMC9059419

[19] M. M. Bos , R. A. J. Smit , S. Trompet , D. van Heemst , and R. Noordam , “Thyroid Signaling, Insulin Resistance, and 2 Diabetes Mellitus: A Mendelian Randomization Study,” Journal of Clinical Endocrinology and Metabolism 102, no. 6 (2017): 1960–1970. 28323940 10.1210/jc.2016-2816

[20] A. Shekarian , S. Mazaheri‐Tehrani , S. Shekarian , et al., “Prevalence of Subclinical Hypothyroidism in Polycystic Ovary Syndrome and Its Impact on Insulin Resistance: A Systematic Review and Meta‐Analysis,” BMC Endocrine Disorders 25, no. 1 (2025): 75. 40102852 10.1186/s12902-025-01896-2 PMC11921581

[21] A. Aktar Karakaya , E. Unal , A. Bestas , and O. Kapcay , “Is Insulin Resistance Present in Children With Subclinical Hypothyroidism?,” Clinical Pediatrics 64 (2025): 99228251333302. 10.1177/00099228251333302 40219792

[22] E. Maratou , D. J. Hadjidakis , A. Kollias , et al., “Studies of Insulin Resistance in Patients With Clinical and Subclinical Hypothyroidism,” European Journal of Endocrinology 160, no. 5 (2009): 785–790. 19141606 10.1530/EJE-08-0797

[23] C. Xu , L. Zhou , K. Wu , et al., “Abnormal Glucose Metabolism and Insulin Resistance Are Induced via the IRE1alpha/XBP‐1 Pathway in Subclinical Hypothyroidism,” Frontiers in Endocrinology 10 (2019): 303. 31156553 10.3389/fendo.2019.00303 PMC6533547

[24] S. Bao , F. Li , L. Duan , J. Li , and X. Jiang , “Thyroid‐Stimulating Hormone May Participate in Insulin Resistance by Activating Toll‐Like Receptor 4 in Liver Tissues of Subclinical Hypothyroid Rats,” Molecular Biology Reports 50, no. 12 (2023): 10637–10650. 37884783 10.1007/s11033-023-08834-2

[25] H. Zhang , Z. Zeng , Y. Liu , et al., “Thyrotropin Exacerbates Insulin Resistance by Triggering Macrophage Inflammation in Subclinical Hypothyroidism,” Experimental and Molecular Medicine 57, no. 6 (2025): 1246–1259. 40523992 10.1038/s12276-025-01478-1 PMC12229657

